# Analysing the role of sleep quality, functional limitation and depressive symptoms in determining life satisfaction among the older Population in India: a moderated mediation approach

**DOI:** 10.1186/s12889-022-14329-9

**Published:** 2022-10-18

**Authors:** Shreya Banerjee, Bandita Boro

**Affiliations:** Centre for the Study of Regional Development, School of Social Sciences, Jawaharlal Nehru University, New Delhi, India

**Keywords:** Sleep quality, Life satisfaction, Older adults, Mental health, Depression, Functional limitation

## Abstract

**Background:**

Life satisfaction (LS), a useful construct in the study of psycho-social well-being, is an important indicator of healthy aging. With a view to investigate whether the improved longevity in India is accompanied by commensurate levels of well-being and contentment among the older adults , this study aimed to examine (1) the association between LS and sleep quality among older Indian adults aged 60 years and above (2) the mediating role of depression that accounts for the association and (3) the moderating role of functional limitation in this mediation.

**Methods:**

Cross-sectional data from the Longitudinal Ageing Study in India (LASI), Wave-1 (2017-18) was used. Pearson’s correlation coefficients were calculated to investigate the pair-wise relationship between sleep quality, depressive symptoms, functional limitation, and LS. Structural Equation Model was employed to analyse the moderated-mediated association between sleep quality and the level of LS.

**Results:**

Sleep quality had a direct effect (β=-0.12) as well as an indirect effect (β=-0.024) via depressive symptoms on LS, accounting for 83.6 and 16.4 per cent of the total effects, respectively. Also, the interaction term between poor seep quality and functional limitation was positive (β = 0.03, p < 0.001) in determining depressive symptoms, suggesting that higher level of functional limitation aggravated the indirect effect of poor sleep quality on LS.

**Conclusion:**

The findings of the study suggested that ensuring both the physical as well as the mental well-being of the population during the life course may confer in later life the desired level of life satisfaction.

## Introduction

With improvement in longevity, India is experiencing a change in its demographic landscape as the proportion of older adults in the total population is gradually increasing. As per the census of India, 2011, older persons aged 60 years or above accounted for 8.6% of the overall population [1]. India has, thus, acquired the label of “an ageing nation”. The share of the older population aged 60 + years is projected to further rise to 19.5% (319 million) by 2050 [2]. Life expectancy at ages 60 and 80 in India have observed considerable improvement and currently stand at 18 and 7 years respectively, projected to rise further to 21 and 8.5 years, respectively by 2050 [2]. While this improved longevity is indicative of an epidemiological achievement of the country, it also poses the challenge of ensuring ‘healthy aging’ to the policy makers. It needs to be investigated whether the longer life, due to improvement in longevity, is accompanied by better levels of well-being and contentment among the older population . Studies have found that greater life satisfaction is highly associated with improved physical and mental health conditions and longevity, therefore, it is considered a universal indicator of successful ageing [3, 4]. In this regard, life satisfaction (LS), a useful construct in the study of psycho-social well-being, is an important indicator of prosperous aging [5, 6].

Life satisfaction, an indicator of happiness, is defined as a cognitive judgment or subjective attitude towards one’s life [7]. It measures the degree of coherence between the desired goals and the actual outcome achieved [8]. Higher life satisfaction is reported when the life conditions are evaluated in line with one’s expectations [9]. Life satisfaction is a component of subjective well-being, where the presence of positive affect and the absence of negative affect are the affective components [10].

The findings of studies on the determinants of life satisfaction are multi-pronged [11–14]. The negative impact of poor sleep quality on life satisfaction has been observed and demonstrated among older adults [15–17]. Sleep problems are highly prevalent among older adults [14, 18]. The strong association between emotion and sleep, which is documented in previous studies, is increasingly recognized as an important area of research [19]. However, the source of dissatisfaction is less likely due to the changes in the structure and pattern of sleep that occur with the aging process but is more likely associated with the physical and the mental health among older adults [18, 20].

Life dissatisfaction is an effective indicator of an individual’s exposure to depression, suicidal tendencies, and other psychiatric illnesses and disabilities [21]. Among these, depression is highly prevalent among older people, coupled with poor sleep quality [22, 23]. Several studies have indicated that having a depressive disorder adversely affects the quality and satisfaction of life among older adults 24,25,26,27. Moreover, sleep quality has been found to be associated with mental health [28, 29]. Empirical evidence shows a negative impact of poor sleep quality and sleep duration on psychological disorders, such as depression, anxiety, and psychosis [30].

In addition to mental health, previous studies have also well documented the association of higher life satisfaction with better physical health [21, 31–33], self-rated health [34], and longevity [4]. The loss of functional capacity at older ages affects the satisfaction of life and influences individuals to such a degree that they moderate their expression of well-being [35]. Life satisfaction and mental health are highly associated with each other, and additionally, self-rated health and limited functionality are significant contributors to depressive symptoms and psychological distress [36]. Living alone and decline in functional health are recognized to have negative impacts on older adults’ life satisfaction [11]. Disability prevents older adults from performing their social roles and daily routines, which subsequently influences their life satisfaction levels [31].

In order to achieve healthy aging in later life, interventions should be developed to enhance positive psychological factors such as life satisfaction and quality of life as well as to reduce mental health symptoms and sleep disturbance [37]. However, unlike in the developed world, there is a lack of studies addressing the factors affecting life satisfaction among older adults in developing societies such as India. In the traditional Asian cultural norms, due to the existence of the traditional joint family system, older adults are supposed to live with their children under the same roof and (or) other family members, which as a result provides social security, emotional and economic support to the older adults [38–40]. But changes in living arrangements, and family structures are affecting the health and life satisfaction of older adults [41, 86]. Moreover, due to the lack of effective social institutions and broad-based pension or social security schemes in developing countries, the factors affecting the life satisfaction of older adults in developing countries might differ from those affecting older population of the developed world  [38, 42].

Given this backdrop, the present study makes an attempt to draw evidence from the data collected by a recent national-level sample survey to shed light on the nature of the linkage between life satisfaction, sleep quality, depressive symptoms, and functional limitation. Specifically, the central objectives of this study are to examine (1) the relationship between LS and sleep quality among older Indian adults aged 60 years and above, (2) the mediating role of depression that accounts for the association, and (3) the moderating role of functional limitation in this mediation. This paper examines the relationship between various covariates of LS among older adults in India on the basis of the following hypothesis: mental health mediates the association between sleep quality and life satisfaction, and this mediation process is moderated by functional limitations.

## Materials and methods

### Data

Data collected through the nationally representative large-scale sample survey, Longitudinal Ageing Study in India (LASI), Wave 1), conducted during 2017-18, has been used for the present study. The LASI adopted a multistage-stratified area probability cluster sampling design and surveyed 42,949 households across all states and UTs of India (except Sikkim), collecting data from a total sample of 72,250 older adults aged 45 and above (including their spouses irrespective of age). The survey collected data on various aspects of older persons’ health and well-being, including but not limited to disease burden, health-seeking behaviour, psycho-social well-being, and socioeconomic security. In addition, the LASI also conducted assessments of the respondents’ physiological, performance-based, anthropometric, and blood-molecular measurements using several internationally validated biomarker tests. The present analysis considers only the respondents aged 60 years or above (n = 31,464; mean age = 67.9 ± 7.5 years). The detailed profile of the study population is presented in Table 1.

**Table 1:** Distribution of the study population (60 years and above) by background characteristics

Background Characteristics		Total	
		Frequency ^a^	%
Sex	Male	15,098	47.5
	Female	16,366	52.6
Age group	Younger olds (60–69 years)	18,974	58.5
	Older Olds (70 years and above)	12,490	41.5
Place of Residence	Rural	20,725	70.6
	Urban	10,739	29.5
Marital Status	Currently Married	20,090	62.1
	Others	11,374	37.9
Social Group^b^	SC	5140	18.9
	ST	5173	8.1
	OBC	11,886	45.2
	Others	9173	27.7
Religion^b^	Muslim	3731	11.3
	Hindu	23,037	82.2
	Others	4695	6.5
Living Arrangement	Alone	1622	5.7
	Others	29,842	94.3
Education	Illiterate (including some with schooling)	17,691	58.8
	Literate (with or without schooling)	13,773	41.2
Work Status^b^	Never worked	8776	26.4
	Currently not working/ unpaid work	13,856	44.5
	currently working (paid)	8824	29.1
Economic Status	Poorest	6484	21.7
	Poorer	6477	21.7
	Middle	6416	21.0
	Richer	6170	19.2
	Richest	5917	16.5
Chronic disease^b^	None	11,525	37.1
	Only one	9491	30.7
	Two or more	10,342	32.1
Impairment^b^	None	28,387	89.6
	Only one	1810	6.4
	Two or more	1126	4.0
Involvement in payment of bills/ settling of financial matters^b^	No	20,422	68.3
	Yes	10,587	31.7
TOTAL		31,464	100.0

Note: ^a^unweighted sample sizes; ^b^ these categories have 0.3, 0.003, 0.03, 0.3, 0.4, 1.4 per cent missing values respectively

Source: authors’ own calculations from Longitudinal Ageing Study in India (LASI), Main Wave I, (2017-18)

### Measures

#### Outcome Variable: life satisfaction

The LASI asked the respondents to rate a set of 5 (affirmative) statements about satisfaction in life on a 7-point Likert scale (1 = strongly disagree, 7 = strongly agree) to gauge their levels of contentment in life. The scale reliability coefficient (Cronbach’s alpha) of 0.90 indicated excellent internal consistency [43]. A composite score (ranging between 1 and 7) was obtained for each individual for the present analysis. The higher the score, the higher would be the level of life satisfaction.

#### Predictor Variable: sleep quality

The frequency of sleep disturbances experienced during the past one month was assessed on a 4-point Likert scale (1 = never, 4 = frequently, i.e., ≥ 5 nights per week), including 5 items in the LASI. The Cronbach’s alpha measured 0.83, suggesting good reliability. A composite score for sleep quality (ranging from 1 to 4) was constructed, a higher score indicating poorer quality of sleep.

#### Mediator Variable: depressive symptoms

The analysis uses the responses of the Composite International Diagnostic Interview- Short Form (CIDI-SF) scale, one of the two internationally validated and comparable tools (the other being the Centre for Epidemiologic Studies Depression (CES-D) scale) employed by the LASI to assess depressive symptoms and diagnose probable major depression [44, 45]. LASI adopted the definition of depression as ‘an extended period of time (at least two weeks) in which a person experiences a depressed mood or loss of interest or pleasure in activities that were once enjoyed [46]. Accordingly, the survey asked three screening questions to filter out those without any or persistent episodes of depressive tendencies. Finally, those who reported having ‘felt sad, blue, or depressed’ (lasting for two weeks or more in a row, all day long/ most of the day, every day/ almost every day) were asked to indicate a ‘yes’=1 or a ‘no’=0 to having 7 different depressive symptoms. The reliability score of 0.70 suggested acceptable internal consistency. A composite score was calculated (ranging between 0 and 7). The higher the score, the greater is the number of depressive symptoms.

#### Moderator variable: functional limitation

The LASI assessed difficulty faced in performing a total of 13 Activities of Daily Living (ADL) due to a physical, mental, emotional, or memory problem. The respondents were asked to indicate a ‘yes’=1 or a ‘no’=0 to having difficulties (that had lasted for more than three months) in each of the activities. The reliability score for the items in the scale was excellent, equal to 0.91. A composite score was calculated (ranging between 0 and 13). The higher the score, the greater the functional limitation.

The items included in each of the measures described above are listed in Table 2.

**Table 2:** Description of Measures included in the Analytical Framework

Measure	Number of items	Scale items	Gradations of each scale item	Range of composite Score	Scale reliability coefficient (Cronbach’s alpha)
Life Satisfaction	Five	In most ways my life is close to ideal’; ‘The conditions of my life are excellent’; ‘I am satisfied with my life’; ‘So far, I have got the important things I want in life’; ‘If I could live my life again, I would change almost nothing”	7 (1 = strongly disagree, 7 = strongly agree)	1–7	*α = 0.90* *(excellent)*
Poor Sleep Quality	Five	Trouble falling asleep, waking up at night and having trouble getting back to sleep, waking too early in the morning and not being able to fall asleep, feeling unrested during the day, and taking a nap during the day	4 (1 = never, 4 = frequently, i.e., ≥ 5 nights per week)	1–4	*α = 0.83* *(good)*
Depressive Symptoms	Seven	Loss of interest, feeling tired, abnormal appetite, trouble concentrating, feeling of worthlessness, thinking about death and trouble falling asleep	2 (0 = no, 1 = yes)	0–7	*α = 0.70* *(acceptable)*
Functional Limitations	Thirteen	Dressing, walking across the room, bathing, eating, getting in or out of bed, using the toilet (including getting up and down), preparing a hot meal (cooking and serving), shopping for groceries, making telephone calls, taking medications, doing work around the house or garden, managing money, such as paying bills and keeping track of expenses, getting around or finding address in unfamiliar place	2 (0 = no, 1 = yes)	0–13	*α = 91* (excellent)

Source: Summarised from Longitudinal Ageing Study in India (LASI), Main Wave I, (2017-18) Questionnaire by the authors

#### Covariates

Based on previous literature on the determinants of Life Satisfaction, five broad domains of covariates have been identified and included in the analysis as controls [4, 11, 25, 36, 42, 47–49]. These domains pertain to demographic factors (age, sex, marital status, religion, social group); social support factor (living arrangement); socioeconomic factors (residence, economic status, education, work status); health conditions (chronic ailments, impairments); and financial empowerment (intra-household involvement in financial matters).

### Statistical analysis

Descriptive statistics (mean and standard deviations) of each of the measures were calculated along with Pearson’s correlation coefficients to investigate the pair-wise relationship between sleep quality, depressive symptoms, functional limitation, and life satisfaction. Mean comparison tests were conducted to examine the inter-group mean differences in the respective measures. The t-statistics of the mean differences were tested for statistical significance by two-tailed p-values.

**Fig. 1:**
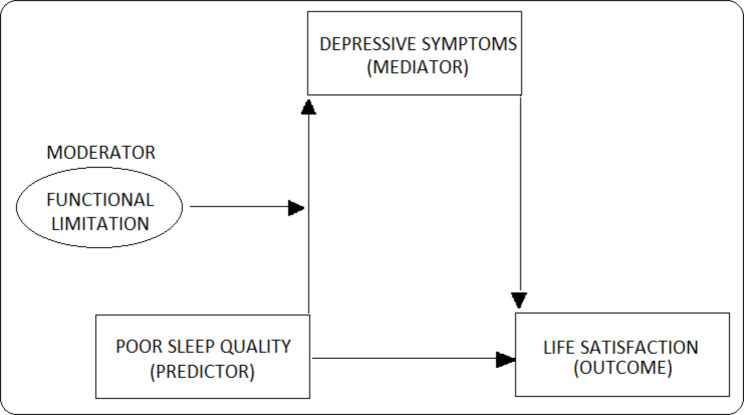
Analytical Framework (Moderated-Mediation)

It is hypothesised that some of the effect of the predictor (sleep quality) on the outcome (life satisfaction), passes through the mediator (depressive symptoms), constituting an indirect effect. Moreover, functional limitation interacts with sleep quality such that the effect of sleep quality on depressive symptoms changes depending on the level of functional limitation (moderator), thereby constituting a conditional indirect effect [50]. The analytical framework of this moderated mediation process is presented in Fig. 1. Structural Equation Model (SEM) was employed to analyse the moderated-mediated association between sleep quality and the level of life satisfaction. The SEM generated path coefficients from two different ordinary least squares (OLS) models; one with depressive symptoms (mediator) as the response variable and the other with life satisfaction (outcome) as the response variable. The covariates were controlled for in both the models. Conditional indirect effects were obtained by multiplying coefficients from the SEMs at three different values of the moderator variable; mean – 1 standard deviation or SD (low moderator), mean (medium moderator), and mean + 1 SD (high moderator). Bootstrap estimates of standard errors and bias-corrected confidence intervals were computed with 5000 repetitions of resampling. The SEM can be expressed in a simplified form as follows:

1 $$m{\rm{ }} = {\rm{ }}{a_0} + {\rm{ }}{a_1}x{\rm{ }} + {\rm{ }}{a_2}w{\rm{ }} + {\rm{ }}{a_3}x*w{\rm{ }} + {\rm{ }}{a_4}{c_1} + {\rm{ }}{a_5}{c_2} \ldots .{\rm{ }} + {\varepsilon _1}$$

2 $$y{\rm{ }} = {\rm{ }}{b_0} + {\rm{ }}{b_1}m{\rm{ }} + {\rm{ }}{b_2}x{\rm{ }} + {\rm{ }}{b_3}w{\rm{ }} + {\rm{ }}{b_4}{c_1} + {\rm{ }}{b_5}{c_2} \ldots .{\rm{ }} + {\varepsilon _2}$$

Where, m = mediator; x = predictor; y = outcome; w = moderator; c_n_ are the covariates; a_n_ and b_n_ are the respective regression coefficients; *ε*_*n*_ are the error terms; b_2_ = direct effect; a_1_* b_1_ = indirect effect; a_1_(b_1_ + a_3_*w) = conditional indirect effect (that varies with varying values of the moderator).

Since the missing values were at random, observations with missing data in categorical variables were excluded from the analysis. Missing values in continuous variables were imputed by the mean of the observed values. Sample weights as provided by the LASI, 2017-18 [87] were applied in the analyses to account for selection probabilities and adjust for non-response. All the statistical analyses were carried out using the software STATA (version 16).

## Results

### Inter-correlations between the model variables

The results of the correlation analysis, presented in Table 3, revealed that poor sleep quality is positively correlated with depressive symptoms. Functional limitation is positively correlated with both poor sleep quality and depressive symptoms. Poor sleep quality, depressive symptoms, and functional limitation are all negatively correlated with life satisfaction. All the inter-correlations were highly statistically significant, albeit being weak or moderate.

**Table 3:** Means, standard deviations, and intercorrelations of the study variables

	Poor Sleep Quality	Depressive Symptoms	Functional Limitation	Life Satisfaction
Poor Sleep Quality	1			
Depressive Symptoms	0.17†	1		
Functional Limitation	0.26†	0.16†	1	
Life Satisfaction	-0.10†	-0.13†	-0.12†	1
Mean	1.8	0.49	2.18	4.75
Std. Dev.	0.75	1.65	3.09	1.52

Note: † p < 0.001

Source: authors’ own calculations from Longitudinal Ageing Study in India (LASI), Main Wave I, (2017-18)

### Mean scores of core model-variables by select covariates

The results of the bivariate analysis of the mean differences between different demographic and socioeconomic groups are presented in Table 4. Female older persons had higher levels of poor sleep quality, depressive symptoms and functional limitations, and a lower level of life satisfaction than the males. Those currently married had greater life satisfaction than those who were not. Older persons living alone had higher levels of depressive symptoms than those living with spouse and/or children or others. The level of functional limitation differed among the illiterate and literate older persons , disfavouring the illiterates. Older persons with at least one impairment had a lower level of life satisfaction compared to those without any. Also, those involved in their intra-household decision-making on financial matters had a better quality of sleep, lower levels of depressive symptoms and functional limitations, and higher life satisfaction than those without such involvement.

**Table 4:** Inter-group mean differences in the study variables by select covariates

Covariates		POOR SLEEP QUALITY		DEPRESSIVE SYMPTOMS		FUNCTIONAL LIMITATION		LIFE SATISFACTION	
		Mean	Mean Difference	Mean	Mean Difference	Mean	Mean Difference	Mean	Mean Difference
Sex	Male	1.71	-0.14†	0.36	-0.07†	1.44	-0.95†	4.84	0.1†
	Female	1.86		0.43		2.39		4.74	
Age group	Younger olds (60–69 years)	1.74	-0.12†	0.38	-0.03	1.38	-1.42†	4.79	0.02
	Older Olds (70 years and above)	1.86		0.41		2.80		4.77	
Place of Residence	Urban	1.76	-0.05†	0.30	-0.14†	1.68	-0.39†	5.00	0.3†
	Rural	1.80		0.44		2.07		4.70	
Marital Status	Currently Married	1.74	-0.13†	0.34	-0.14†	1.51	-1.19†	4.86	0.21†
	Others (widowed/ divorced/ separated/ never married)	1.87		0.49		2.70		4.65	
Social Group	SC/ST	1.74	-0.7†	0.34	-0.08†	1.86	-0.11**	4.66	-0.19†
	Others (incl. OBC)	1.81		0.42		1.97		4.85	
Religion	Hindus	1.81	0.8†	0.42	0.1†	1.98	0.17†	4.78	-0.04
	Minority religious groups	1.73		0.32		1.81		4.82	
Living Arrangement	Alone	1.89	0.11†	0.61	0.23†	2.31	0.40†	4.28	-0.54†
	Not alone	1.78		0.38		1.91		4.82	
Education	Illiterate	1.83	0.11†	0.44	0.11†	2.44	1.15†	4.59	-0.45†
	Literate (with or without schooling)	1.72		0.33		1.29		5.04	
Work Status	Engaged in paid work	1.65	-0.19†	0.35	-0.06***	0.96	-1.36†	4.75	-0.05*
	Others	1.84		0.41		2.32		4.80	
Economic Status	Poor	1.78	-0.01	0.41	0.02	2.15	0.27†	4.57	-0.27†
	Non-poor	1.79		0.39		1.88		4.84	
Chronic disease	None	1.63	-0.24†	0.28	-0.18†	1.50	-0.68†	4.79	0.01
	At least one	1.87		0.46		2.18		4.78	
Impairment	None	1.77	-0.19†	0.35	-0.46†	1.77	-1.96†	4.82	0.41†
	At least one	1.96		0.81		3.73		4.41	
Involvement in payment of bills/ settling of financial matters	No	1.85	0.17†	0.42	0.07†	2.47	1.55†	4.74	-0.15†
	Yes	1.68		0.35		0.92		4.89	

*Note*: † p < 0.001, *** p < 0.01 ** p < 0.05 and * p < 0.1

Source: authors’ own calculations from Longitudinal Ageing Study in India (LASI), Main Wave I, (2017-18)

### Mediation effect of depressive symptoms on the association between sleep quality and life satisfaction, moderated by functional limitation

**Fig. 2:**
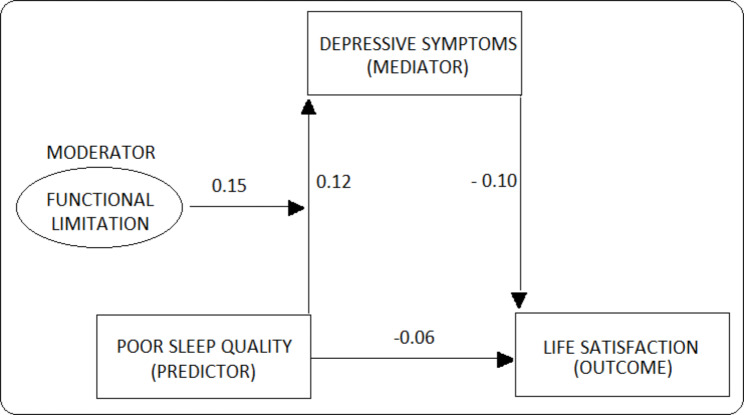
Standardised coefficients of the moderated mediation model

The results of the regression analysis, presented in Table 5, showed that poor sleep quality had negative effect (β=-0.12, p < 0.001) on life satisfaction. Poor sleep quality also had a positive effect (β = 0.27, p < 0.001) on depressive symptoms, which in turn had a negative effect (β=-0.09, p < 0.001) on life satisfaction. Thus, sleep quality had a direct effect (β=-0.12) as well as an indirect effect (β=-0.024) via depressive symptoms on life satisfaction, accounting for 83.6 and 16.4% of the total effects, respectively (Table 5). The standardised coefficients of the moderated mediation analysis have been presented in Fig. 2. Also, while functional limitation had a negative effect on life satisfaction (β=-0.029, p < 0.001), its effect on depressive symptoms was statistically insignificant. However, the interaction term between poor sleep quality and functional limitation was positive and statistically significant (β = 0.03, p < 0.001), suggesting that a higher level of functional limitation aggravated the effect of poor sleep quality on depressive symptoms. This conditional indirect effect was calculated and presented in Table 6 at three different values of functional limitation- low (mean-std dev), medium (mean), and high (mean + std dev).

Living arrangement, place of residence, work status, chronic morbidity, impairment, and involvement in financial matters showed a statistically significant effect on depressive symptoms. Besides, gender, marital status, social group, place of residence, literacy, economic status, and impairment were statistically significant determinants of life satisfaction.

**Table 5:** Results of the moderated mediation analysis

Predictors	Coeff.	Robust SE	[95% Conf. Interval]	
**Outcome: Depressive Symptoms**				
*Poor sleep quality*	*0.2689†*	*0.0275*	*0.2149*	*0.3228*
*Functional Limitation*	*0.0003*	*0.0166*	*-0.0329*	*0.0322*
Poor sleep quality * Functional Limitation	*0.0338†*	*0.0086*	*0.0170*	*0.0507*
Age	-0.0280	0.0300	-0.0869	0.0309
Age squared	0.0001	0.0002	-0.0003	0.0005
Female	0.0180	0.0420	-0.0643	0.1003
Currently Married	-0.0403	0.0397	-0.1182	0.0375
SC/ ST	-0.0498	0.0369	-0.1222	0.0226
Hindu	0.0031	0.0385	-0.0724	0.0786
Living alone	0.1669**	0.0818	0.0067	0.3272
Rural	0.1475†	0.0319	0.0849	0.2101
Illiterate	0.0012	0.0363	-0.0701	0.0724
Currently working (paid)	0.0754**	0.0356	0.0057	0.1451
Poorest	0.0062	0.0361	-0.0646	0.0771
At least one chronic ailment	0.0651*	0.0360	-0.0055	0.1356
At least one impairment	0.3753†	0.0726	0.2329	0.5176
Involved in financial matters	0.0661*	0.0344	-0.0013	0.1335
**Outcome: Life Satisfaction**				
*Depressive Symptoms*	*-0.0898†*	*0.0095*	*-0.1084*	*-0.0713*
*Poor Sleep quality*	*-0.1220†*	*0.0213*	*-0.1637*	*-0.0803*
*Functional Limitation*	*-0.0293†*	*0.0069*	*-0.0428*	*-0.0158*
Age	0.0121	0.0302	-0.0470	0.0712
Age squared	0.0000	0.0002	-0.0004	0.0004
Female	0.1250†	0.0364	0.0537	0.1963
Currently Married	0.0923**	0.0385	0.0169	0.1677
SC/ ST	-0.1621†	0.0317	-0.2242	-0.0999
Hindu	-0.0122	0.0359	-0.0826	0.0582
Living alone	-0.5138†	0.0845	-0.6794	-0.3481
Rural	-0.1335***	0.0434	-0.2186	-0.0485
Illiterate	-0.3761†	0.0384	-0.4513	-0.3008
Currently working (paid)	-0.0407	0.0339	-0.1071	0.0258
Poorest	-0.2106†	0.0391	-0.2873	-0.1340
At least one chronic ailment	-0.0304	0.0329	-0.0948	0.0341
At least one impairment	-0.4022†	0.0651	-0.5297	-0.2747
Involved in financial matters	0.0353	0.0368	-0.0368	0.1074
**Fit Statistics**: Standardized root mean squared residual (SRMR) 0.000 Coefficient of determination (CD) 0.124				

Note: † p < 0.001, *** p < 0.01 ** p < 0.05 and * p < 0.1

Source: authors’ own calculations from Longitudinal Ageing Study in India (LASI), Main Wave I, (2017-18)

**Table 6:** Total, direct, indirect and conditional indirect effects

Effects	Coef.	Std. Err.	[95% Conf. Interval]	
*Total*	-0.1461†	0.0209	-0.1872	-0.1051
*Direct*	-0.122†	0.0213	-0.1637	-0.0803
*Indirect*	-0.0241†	0.0036	-0.0312	-0.0171
*Conditional Indirect*		Bootstrapped Std. Error.	Bias corrected [95% CI]	
*M-SD*	-0.0336	0.0040	-0.0417	-0.0266
*M*	-0.0066	0.0054	-0.0173	0.0032
*M + SD*	0.0205	0.0105	-0.0002	0.0399

Note: † p < 0.001, ** p < 0.05

Source: authors’ own calculations from Longitudinal Ageing Study in India (LASI), Main Wave I, (2017-18)

### Robustness check

In order to verify whether the moderated mediation relationship between poor sleep quality and life satisfaction is robust to specification changes in our model, we conducted a sensitivity analysis [51] by estimating a set of regressions where the outcome variable was regressed on a set of core variables (included in all the regressions) and every possible combination of certain testing/ non-core/ secondary variables. A total of 4096 (= 2^12^) regression models were estimated for each of the two outcomes of the structural equation model of Table 4, i.e., depressive symptoms and life satisfaction. For the model with depressive symptoms as the outcome, poor sleep quality, functional limitation, and their interaction (multiplicative) term were defined as the three core variables, while for the model with life satisfaction as the dependent variable, depressive symptoms, poor sleep quality, and functional limitation constituted the core variables. All the predictors in Table 4 were considered secondary, except the variables age and age-squared, which were always included in all the regressions. Thus, twelve variables (sex, marital status, social group, religion, living arrangement, place of residence, education, work status, wealth quintile, chronic disease, impairment) were regarded as non-core. The results of the sensitivity analysis are presented in Table 7.

**Table 7:** Summary statistics of sensitivity analysis for checking robustness of the model

Outcome: Depressive Symptoms											
**Core variables**	**Max** ^i^	**Min** ^ii^		**Mean** ^iii^		**Average Std. Dev.** ^iv^	**Percentage Significant** ^v^	**Percentage positive** ^vi^	**Percentage negative** ^vii^	**Average t-value** ^viii^	**No. of Obs.** ^ix^
Poor Sleep Quality	0.245	0.217		0.231		0.014	100	100	0	16.23	4096
Functional Limitation	0.025	0.007		0.016		0.008	53.4	100	0	2.045	4096
Poor Sleep Quality X Functional Limitation	0.024	0.021		0.022		0.004	100	100	0	6.369	4096
**Testing variables**											
Female	0.013	-0.079		0.031		0.019	38	93	7.0	1.669	2048
Currently Married	-0.043	-0.103		-0.078		0.02	100	0	100	3.99	2048
SC/ ST	-0.039	-0.087		-0.065		0.018	100	0	100	3.56	2048
Hindu	0.071	0.04		0.056		0.019	100	100	0	2.933	2048
Living alone	0.203	0.119		0.16		0.039	100	100	0	4.107	2048
Rural	0.132	0.087		0.108		0.018	100	100	0	5.884	2048
Illiterate	0.058	-0.028		0.015		0.019	17	77	23	1.057	2048
Currently working (paid)	0.08	0.017		0.044		0.02	63	100	0	2.203	2048
Poorest	0.04	0.004		0.021		0.021	0	100	0	1.024	2048
At least one chronic ailment	0.101	0.063		0.084		0.018	100	100	0	4.668	2048
At least one impairment	0.296	0.278		0.287		0.03	100	100	0	9.425	2048
Involved in financial matters	0.089	0.041		0.066		0.019	100	100	0	3.398	2048
**Outcome: Life Satisfaction**											
**Core variables**											
Depressive Symptoms	-0.089		-0.100	-0.094	0.006		100	0	100	16.690	4096
Poor Sleep Quality	-0.112		-0.139	-0.124	0.012		100	0	100	10.625	4096
Functional Limitation	-0.028		-0.050	-0.038	0.003		100	0	100	12.285	4096
**Testing variables**											
Female	0.170		-0.076	0.053	0.019		66	74	26	3.598	2048
Currently Married	0.185		0.036	0.121	0.019		99.6	100	0	6.354	2048
SC/ ST	-0.089		-0.215	-0.142	0.018		100	0	100	7.972	2048
Hindu	0.017		-0.049	-0.015	0.019		8.0	19	81	0.921	2048
Living alone	-0.390		-0.522	-0.446	0.038		100	0	100	11.720	2048
Rural	-0.137		-0.282	-0.210	0.018		100	0	100	11.792	2048
Illiterate	-0.281		-0.423	-0.348	0.018		100	0	100	19.322	2048
Currently working (paid)	-0.048		-0.168	-0.101	0.020		100	0	100	5.127	2048
Poorest	-0.179		-0.265	-0.221	0.020		100	0	100	10.849	2048
At least one chronic ailment	0.068		-0.055	-0.006	0.018		27	36	64	1.395	2048
At least one impairment	-0.246		-0.279	-0.262	0.030		100	0	100	8.798	2048
Involved in financial matters	0.126		-0.034	0.045	0.019		59	89	11	2.555	2048

*Notes*: ^*i*^*maximum point estimate*, ^*ii*^*minimum point estimate*, ^*iii*^*average point estimate*, ^*iv*^*average standard deviation of the point estimates*, ^*v*^*share of regressions (in %) where point estimate was significant at 0.05 level*, ^*vi*^*share of regressions (in %) with a positive point estimate (may or may not be significant)*, ^*vii*^*share of regressions (in %) with a negative point estimate (not necessarily significant)*, ^*viii*^*average t-value over all regressions*,^*ix*^*total number of estimated regression models*

The sensitivity analysis revealed that the results remained largely unaffected when one or more predictors were omitted, thereby confirming the robustness of our proposed model. In the case of the model with depressive symptoms as the outcome, the coefficients of the core variables were positive in 100% of the regressions, therefore indicating no instance of sign change in any combination of the testing variables. Similarly, there was zero instance of sign change in the coefficients of the core variables in the model with life satisfaction as the outcome, where the sign was negative in 100% of the regression estimates. The effect of poor sleep quality on depressive symptoms was statistically significant (at 0.05 significance level) in 100% of the cases. Functional limitation was a statistically significant predictor of depression in only 53.4% of the cases. However, the interaction term between poor sleep quality and functional limitation was statistically significant in 100% of the cases. In the model with life satisfaction as the outcome variable, on the other hand, each of the three core predictor variables were statistically significant at 0.05 level 100% of the time in determining life satisfaction among older adults in India.

## Discussion

This study explored the associations between life satisfaction and sleep quality and whether depression mediated this association. The study also examined the moderating effect of functional limitation on the association between sleep quality and depression. In this study, it was found that poor sleep quality had a negative effect on life satisfaction. Furthermore, we found that poor sleep quality had a positive effect on depression, which in turn had a negative effect on life satisfaction among older adults aged 60 or above in India. Therefore, sleep quality had both direct and indirect effects on life satisfaction among older adults. The indirect effect was moderated by functional limitation, and a stronger effect was observed in older adults with a higher level of functional limitations. Thus, functional limitation aggravated the effect of poor sleep quality on depressive symptoms. Therefore, both our hypotheses are supported by the findings of this study.

The findings of this study that poor sleep quality was associated with a higher level of depression fall in line with previous studies on older adults [17, 52, 53]. On the other hand, studies have also explored the mediating role of depression in the association between sleep quality and quality of life which is similar to the construct of life satisfaction [54]. Short sleep duration and poor sleep quality at night may lead to daytime tiredness, which increases adverse events and emotions and eventually predisposes individuals to a risk of depression [55]. Moreover, poor sleep quality has been associated with specific health behaviours to cope with stress, such as smoking and drinking alcohol, misuse of medications, and overeating which might increase the risk of depression [56–58]. The mediation analyses also indicated a significant mediating effect of mental health on the association between sleep quality and life satisfaction. Meanwhile, a study in China has also demonstrated that short sleep duration and poor sleep quality were inversely associated with life satisfaction and that the associations were partially mediated by the effects of depression [12]. Poor sleep quality affects cognitive and physical function, interaction with family and social relationships, and self-perception of health [59] which in turn can lead to depression. Therefore, poor sleep quality might reduce the life satisfaction of older adults by increasing mental health problems.

Our study also found the association of some of the covariates with life satisfaction to be statistically significant. Life satisfaction was found to be higher for older female adults than males. Researchers have argued that the tendency to report themselves happy is often higher for women than men, as women exhibit a higher capacity to express their emotions [66, 67]. Another study has found that women’s well-being is influenced by education, marital status, and social networks, but men’s happiness depends on occupation status to a large extent [68]. Further studies need to be carried out to understand the gender differential in life satisfaction. Also, older adults belonging to ST/SC social groups had a negative association with life satisfaction which can be a reflection of their social marginalisation [88]. ‘Currently married’ marital status had a positive association with life satisfaction. “Many activities are couple-companionate, undertaken as a couple, with other couples”[60]. Also, the availability of a spouse presumably gives both emotional and economic support.

Older adults living in rural areas had a negative association with life satisfaction. Social welfare programs, pension schemes, and healthcare services are better available in urban areas than in rural areas which might cause lower life satisfaction among older adults living in rural areas [25, 61]. Moreover, socioeconomic factors like illiteracy and poor income of older adults were also negatively associated with life satisfaction. Education and well-being are positively associated as higher income level, productivity, and social status are achieved through education [62]. A person’s happiness and well-being improves with high family income compared to those with lesser family income [63]. Also, an older person with a secure feeling about money and freedom of choice in the present and future has higher life satisfaction [64]. Moreover, older adults with poor income are unable to meet their health expenses for their physical and mental needs, which in turn becomes more stressful for them [65].

The moderated mediation analyses indicated that functional limitation, i.e., ADL moderated the strength of mediating effect of mental health on the association between sleep quality and life satisfaction. Previous studies found that depressive symptoms adversely affect the quality of life, which is a similar construct of life satisfaction through its association with functional limitation, physical health, and mortality [69]. Additionally, limited functionality due to disability exerts influence on psychological well-being, which can subsequently lead to depressive symptoms and psychological distress [36].Individuals with poor mental health would engage in a low-levels of physical activity which would lead to a functional decline and eventually would cause more stress regarding their health status [70], which would further negatively affect the quality of life. Moreover, older adults with functional limitations can be a burden to their family or caregivers which might compromise healthy familial relationships, which in turn may negatively impact the older adults’ life [13, 71]. Also, older adults with a disability are unable to perform social roles and daily routines, which negatively impacts their level of life satisfaction [31].

Additionally, living arrangement, place of residence, work status, chronic morbidity, impairment and involvement in financial matters were found to be statistically significant determinants of depressive symptoms. Older adults who resided in rural areas had a positive association with reporting depressive symptoms. Rural older adults may be overburdened economically to manage their daily living expenses as they are mostly engaged in informal jobs and farming which has no social security and pension schemes [72]. Also, currently working older adults had a positive association with depression. Certain socio-cultural contexts and norms favour retirement as a socially accepted positive status. Thus, retired individuals are more valued than those who still work, which might explain depressive symptoms among working older adults[73]. Moreover, engaging in a job with no optimal conditions or an unsatisfactory job can possibly lead to depression [74].

An interesting finding of our study is that involvement in financial matters was positively associated with reporting depressive symptoms among older adults. This is in contrast to some other studies that have found that financial empowerment or autonomy increases the ability of the adults to take better control of their health and well-being even in their later life [75, 76]. The burden of meeting daily needs even at an older age might lead to depression among older adults. Besides, older adults with a chronic disease or multimorbidity were more susceptible to depression. A chronic disease might lead to loss of functional ability, loss of independence, and negative effects on the inter-personal relationship, ultimately leading to depression [77–79]. Additionally, the presence of one or more impairments was positively associated with depression. Physical and mental impairments lead to dependency on others in terms of self-care and other basic needs, restriction in mobility, low social interaction; hence it may ultimately affect an older persons’ psychological well-being [80, 81].

We also found a positive association of the ‘living alone’ status of older adults with reporting depression, consistent with findings of previous studies that showed older adults living alone had higher odds of depression than those living with their spouses and/ or children [78, 82–84]. Contrastingly, it has also been found that conflicts within the family might lead to feelings of loneliness, which is a risk factor of depression; hence living with family might not always necessarily be a protective factor against depression [85].

The current study is not without limitations. Firstly, due to the cross-sectional design, causal inferences cannot be drawn from this study. Secondly, the study, due to being based on self-reported data, is constrained by the subjectivity of perception and reporting bias. Hence, longitudinal studies and research using objective information about the respective indicators are better suited for analysing cause and effect. Despite these limitations, our study makes a modest attempt to add to the existing pool of literature on the determinants of life satisfaction in later life. Also, the study draws evidence from a nationally representative sample of older adults, which adds to its strength. The findings of the study revealed that successful ageing can be achieved by working on different pathways through which sleep quality and mental and physical health determine the level of life satisfaction, as was elicited in our analysis. Understanding the predictors of life satisfaction may have important implications for future health outcomes, such as the development of chronic medical conditions and other mental health conditions.

## Conclusion

Effective designing of the welfare programmes, policies, and regulations for older adults warrants a better understanding of the relationship between people’s individual characteristics and their perceptions of life satisfaction. Depression should be diagnosed and treated early in order to reduce its adverse effects on life satisfaction. Older adults with functional limitations should be able to access affordable assistive technology, disabled-friendly housing and public spaces, etc., to have the desired level of independence and a sense of dignity in old age. Finally, an effective and efficient social security system is paramount to ensuring successful ageing.

## Data Availability

The data (Longitudinal Ageing Study in India, Wave-1) used for the present analysis is freely available for academic researchers and can be requested from here: https://www.iipsindia.ac.in/content/data-request.

## List of abbreviations

ADL: Activities of Daily Living
CES-D: Centre for Epidemiologic Studies Depression
CIDI-SF: Composite International Diagnostic Interview- Short Form
LASI: Longitudinal Ageing Study in India
LS: Life Satisfaction
OLS: Ordinary Least Squares
SD: Standard Deviation
SEM: Structural Equation Model
